# HIV-free survival and morbidity among formula-fed infants in a prevention of mother-to-child transmission of HIV program in rural Haiti

**DOI:** 10.1186/1742-6405-8-37

**Published:** 2011-10-12

**Authors:** Louise C Ivers, Sasha C Appleton, Bingxia Wang, J Gregory Jerome, Kimberly A Cullen, Mary C Smith Fawzi

**Affiliations:** 1 Division of Global Health Equity, Brigham and Women's Hospital, Boston, MA, USA; 2 Partners In Health, Boston, MA, USA; 3 Department of Global Health and Social Medicine, Harvard Medical School, Boston, MA, USA; 4 Massachusetts General Hospital, Boston, MA, USA; 5 Zanmi Lasante, Cange, Haiti

**Keywords:** HIV, prevention of mother-to-child transmission, breastfeeding, formula, resource-poor setting, Haiti

## Abstract

**Background:**

Partners In Health (PIH) works with the Ministry of Health to provide comprehensive health services in Haiti. Between 1994 and 2009, PIH recommended exclusive formula feeding in the prevention of mother-to-child transmission (PMTCT) of HIV program and provided support to implement this strategy. We conducted this study to assess HIV-free survival and prevalence of diarrhea and malnutrition among infants in our PMTCT program in rural Haiti where exclusive formula feeding was supported.

**Methods:**

We reviewed medical charts of PMTCT mother-infant pairs at PIH between November 2004 and August 2006 through a retrospective longitudinal study and cross-sectional survey. We performed household surveys for each pair and at control households matched by infant's age and gender.

**Results:**

254 mother-infant pairs were included. 15.3% of infants were low birth weight; most births occurred at home (68.8%). 55.9% of households had no latrine; food insecurity was high (mean score of 18; scale 0-27, SD = 5.3). HIV-free survival at 18 months was 90.6%. Within the cohort, 9 children (3.5%) were HIV-infected and 17 (6.7%) died. Community controls were more likely to be breastfed (P = 0.003) and more likely to introduce food early (P = 0.003) than PMTCT-program households. There was no difference in moderate malnutrition (Z score ≤ 2 SD) between PMTCT and community groups after controlling for guardian's education, marital status, and food insecurity (OR = 1.05; 95% CI: 0.67, 1.64; P = 0.84). Diarrhea was 2.9 times more prevalent among community children than PMTCT infants (30.3% vs. 12.2%; P < 0.0001).

**Conclusions:**

In a PIH-supported program in rural Haiti that addressed socioeconomic barriers to ill-health, breast milk substitution was safe, acceptable and feasible for PMTCT for HIV-infected women choosing this option.

## Introduction

It is estimated that more than 33.3 million people are living with HIV/AIDS worldwide, 2.5 million of whom are children [1]. The majority of these HIV-infected children are infected through mother-to-child transmission. Infants born to HIV-infected mothers are at risk for acquiring HIV infection *in utero*, at parturition, and in early life through breast milk. Although antiretroviral therapy (ART) during breastfeeding can substantially reduce the risk of transmission [2], avoidance of breastfeeding remains the only way to ensure prevention of mother-to-child transmission of HIV postnatally. In resource-rich settings, universal HIV testing and counseling, ART, and complete avoidance of breastfeeding have reduced of the risk of mother-to-child transmission to less than 2% [3]. Recent studies from Botswana and Kenya have demonstrated efficacy of the strategy that continues ART during breastfeeding, reducing the risk of transmission to 1.1% and 4.2% at 1 and 6 months, respectively [4]. However, both studies have also shown some remaining risk of HIV transmission during breastfeeding, albeit small [5].

In resource-poor settings, lack of affordable formula and clean water with which to prepare formula exposes immunologically immature infants to life-threatening infections when formula-fed; this may negate the benefits of bottle feeding to prevent HIV transmission. Until November 2009, the World Health Organization (WHO) guidelines recommended that infants born to HIV-infected mothers be exclusively breastfed for six months followed by rapid weaning in settings where it was unlikely that exclusive formula feeding was "acceptable, feasible, affordable, sustainable and safe" [6,7]. In July 2010, these guidelines were formally changed to recommend combination ART to pregnant HIV-infected women through delivery and to continue during the breastfeeding period [8].

To date, most data evaluating exclusive formula feeding for infants born to HIV-infected mothers have been through randomized clinical trials and not as part of a programmatic evaluation [9,10]. Between 1996 and 2010, the non-governmental organization Partners In Health (known as Zanmi Lasante in Haitian Kreyol) recommended formula feeding for infants born to women with HIV infection in its healthcare programs in Haiti. Despite the poor social situation in which most women and their families were living, Zanmi Lasante (ZL) provides support to ensure that breast milk substitution could be acceptable, feasible and safe--and provides a robust supply of formula to mothers free of charge and by prescription.

The objective of this study was to assess HIV-free survival among a cohort of infants born to HIV-positive women enrolled in a community-based prevention of mother-to-child transmission of HIV (PMTCT) program in rural Haiti between November 1, 2004 and August 31, 2006. We also compared the prevalence of diarrhea and malnutrition in this cohort of infants with a cohort of infants of unknown HIV exposure or infection status, matched according to age, gender, and community.

## Methods

### Setting

HIV/AIDS remains a leading cause of morbidity and mortality in Haiti. In 2009, there were as many as 120,000 individuals infected with HIV in Haiti, including 12,000 pediatric cases [11]. The prevalence of HIV infection among adults is 1.9% [12]. Zanmi Lasante operates a network of 15 sociomedical complexes in rural Haiti's Central Plateau and Artibonite departments in collaboration with the Haitian Ministry of Health. This study was conducted in Haiti's Central Plateau, a region home to approximately 550,000 inhabitants. The population is mostly comprised of subsistence farmers; over half of the adults are illiterate and food insecurity is high [13,14]. ZL supports mothers with HIV and their infants through a community-based PMTCT program. At the time of this study, women who enrolled in the PMTCT program were offered combination ART from week 28 of pregnancy, or earlier, if needed, for the mother's health (i.e. if the mother's CD4 count is less than 350 cells/mm3). The program strongly encouraged formula feeding to prevent the transmission of HIV from mother-to-child and supported non-breastfeeding, HIV-positive women by providing formula free of charge, by prescription, as well as social support. In all other circumstances, ZL encouraged exclusive breastfeeding for six months as the healthiest option for mother and child. For women with HIV who chose not to breastfeed, the PMTCT social support program provided tools and comprehensive education for preparation of clean water in order to safely prepare formula, and accompaniment by means of frequent visits of a community health worker. The role of community health workers in providing support in PIH programs has been described previously [15,16]. After 9 months of formula feeding, children were transitioned off prescription formula and enrolled in a food assistance program to support transition to solid foods until 18 months of age. HIV-exposed infants were prescribed co-trimoxazole prophylaxis beginning at four weeks of age. This was discontinued if and when HIV testing confirmed the infant to be HIV negative. Most infants were no longer eligible for co-trimoxazole prophylaxis by this criterion at the time of the household survey (below).

### Study design

We employed two study designs. First, we conducted a longitudinal study to measure the 18-month cumulative probability of HIV-free survival among children born to women enrolled in the ZL PMTCT program between November 1, 2004 and August 31, 2006. By choosing these dates, infants were 18 months of age at the time of the study, allowing HIV diagnostic testing protocols to be complete. Mother-child pairs were eligible for inclusion in this cohort analysis if the mother enrolled for care in the ZL PMTCT program before or at the time of delivery of her child. Infants and mothers who enrolled for care after delivery of the infant were excluded as we did not aim to measure HIV free survival amongst mother-infant pairs that had not received any antiretroviral prophylaxis.

Second, we conducted a cross-sectional analysis to compare the prevalence of diarrhea and malnutrition between the children enrolled in the PMTCT program with a comparison group in the community. The comparison group included children that were not in the PMTCT program and that were matched according to age, gender, and community. For the cross-sectional analysis, children enrolled in the PMTCT program were eligible to be included if they were alive and at least 18 months of age at the time of study.

Trained Haitian Kreyol-speaking enumerators performed a survey at the household of every eligible child in the PMTCT cohort. Community controls were then selected according to matching criteria using a random walk method [17]. In households chosen as potential controls, the female head of household was asked if she had at least one live birth within 6 months before or after the date of birth of the program child just visited. If "yes", the mother was invited to participate in the study and complete an interview. If no child was born within this time frame, then the interviewer continued on to the next home and repeated the procedure. If the mother of the child was no longer alive but the child fulfilled the matching criteria, the present guardian of the child was invited to complete the interview.

### Zanmi Lasante PMTCT program

Mothers in the Zanmi Lasante PMTCT program were confirmed to be positive for HIV infection and treated to prevent HIV transmission using a clinical protocol. Some women were given azidothymidine (AZT), lamivudine (3TC), nevirapine (NVP) twice daily starting at 28 weeks of gestation until delivery or single-dose NVP at delivery if presenting for the first time. Others received AZT only starting at 28 weeks of gestation. The variation in treatment was due to the fact that initially the Haiti Ministry of Health national protocol called for use only of AZT but some physicians chose a more conservative treatment with three drugs regardless of this protocol. Over time, the three-drug regimen became standard of care. If the mother received single-dose NVP at delivery, she then received 14 days of AZT and 3TC postpartum. Infants born to women enrolled in the ZL PMTCT program before 36 weeks gestation were given 4 mg/kg of AZT twice daily for 7 days after delivery. Infants born to women who presented after 36 weeks gestation were given a single-dose of NVP at the time of birth followed by 4 mg/kg of AZT for duration of 6 weeks.

PMTCT program mother-infant pairs were followed up during monthly clinic visits until the infant was 18 months of age. HIV infection in program children was tested for using standardized protocols to test for HIV RNA or DNA using specimens for dried blood spots at the first and fourth months of life. HIV status was confirmed with a series of rapid antibody assays at birth, 6-, 12- and 18-months of age [18]. In the postpartum period, mothers were encouraged to exclusively formula feed for six months and were provided with the tools for the preparation of clean water, education and accompaniment. Formula was provided free of charge to mothers in the program by monthly prescription for nine months. During the period of time in which solid food was introduced, food support was provided to facilitate mothers that could not afford to purchase nutritious foods. This support was in the form of dry rations to the family or a cash transfer monthly until the infant was of age of 18 months.

### Data Collection and Definitions

The household interview used to collect data for PMTCT and community infants and their mothers or guardians was conducted using a standardized questionnaire. Additional data for PMTCT infants and their mothers were collected during a clinical chart abstraction. All interviews were carried out in Haitian Kreyol by native Kreyol speakers trained specifically according to the study protocol.

A child was classified as being HIV-infected if he or she tested positive for HIV by direct antigen testing (HIV RNA or HIV DNA) before 18 months of age or by rapid antibody testing at 18 months of age or older. Discordant or indeterminate test results were confirmed by additional testing and a review of the child's clinical history by a clinician. A child was determined to be HIV-negative if all assays performed before and after 18 months of age yielded a negative result for HIV. HIV-free survival was defined as being alive and having a confirmed negative HIV status over the follow-up period. Thus, the clinical endpoints for calculating HIV-free survival included time to HIV infection or time to death. In this analysis, we used the date of the first event (either HIV infection or death if the child experienced both events) as the event date. If a child died and the date of death was not known, we imputed it as the midpoint from the last date the child was known to be alive and the date of the interview.

Information collected included household demographic information, access to health services, infant's gender, birth weight (when available), premature birth (defined as the mother's report of birth before 37 weeks gestation), Caesarean delivery, birth location (home versus institutional), birth order, whether the infant was ever breastfed, mother's age at delivery, mother's antiretroviral regimens before delivery, type of antenatal care received by the mother, infant feeding practices, and age of weaning. An assessment of adherence to exclusive infant feeding (either breastfeeding or formula feeding) was made during the study interview using a series of questions, as well as by reviewing narrative information taken from the infant's clinical chart. Data was also collected on infants' age in months, weight and prevalence of diarrhea (defined as 3 or more watery stools in a 24-hour period) within the two weeks preceding the date of the interview. Primary morbidity endpoints were diarrhea and nutritional status. Household food security was measured using the Household Food Insecurity Access Scale (HFIAS) [19], and poverty score was measured using a scale previously validated in Haiti [20,21].

### Statistical Analysis

The Kaplan-Meier method was used to measure the 18-month cumulative probability of HIV-free survival among children in the ZL PMTCT cohort. Cox proportional hazards regression was used to identify associations between HIV-free survival and potential predictors in both univariate and multivariate analyses. Children who were unable to be located for an interview or who were lost to follow up were censored at the age of 18 months. Baseline variables that were statistically different at the P = 0.10 level were included in the multivariate analysis as potential confounding factors. Hazard ratios with corresponding 95% confidence intervals (CI) for HIV-free survival were presented.

In the cross sectional analysis, mother and infant characteristics collected at the time of interview were compared between the ZL PMTCT cohort and the community cohort by using a t-test for continuous variables and the Chi-square test for categorical variables. To examine the difference of the nutritional status between the two groups, weight-for-age Z scores were analyzed for overall population and by age stratum (12-23, 24-35, 36-47, and 48-60 months) through a t-test for mean Z scores and logistic regression for the proportions of < -2SD and -3SD. Additionally, a multivariate logistic model was constructed to evaluate the impact of the interaction effect on the proportion of < -2SD. To compare the prevalence of diarrhea between ZL PMTCT infants and the community infants, univariate and multivariate analyses were performed through logistic regression models.

Analyses were conducted using SAS software (version 9.1 or higher, SAS Institute Inc, Cary, North Carolina, USA). Two-sided p-values < 0.05 were considered statistically significant.

Ethics committee approval was obtained for this study from Brigham and Women's Hospital, as well as Zanmi Lasante.

## Results

**Figure 1:**
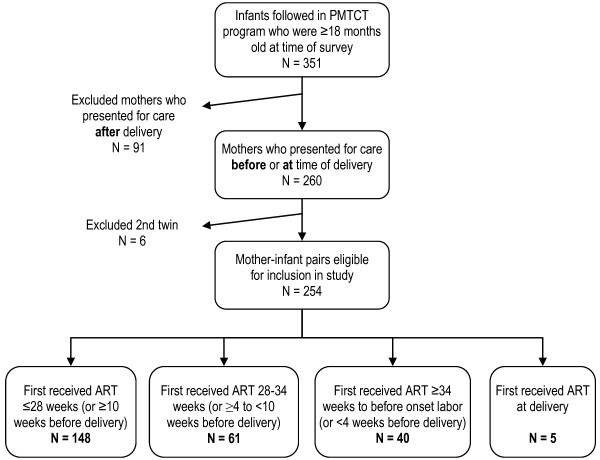
**Enrollment in an analysis of a Zanmi Lasante PMTCT program in rural Haiti**. ART = Antiretroviral therapy.

From November 1, 2004 to August 31, 2006, 351 HIV-positive women and their infants presented for care at Zanmi Lasante (Figure 1). Mothers who presented after delivery (N = 91) were ineligible for inclusion in the study. A total of 260 mothers were enrolled in the PMTCT program. After excluding the second infant in 6 sets of twins, there was an effective sample size of 254 mother-infant pairs.

Baseline characteristics of the study cohort are shown in Table 1. Of the 254 infants, 42.9% were girls, 15.3% were low birth weight, 5.4% were born prematurely and most of the births occurred at home (68.8%). Most women, 58.3% (N = 148), were started on ART at 28 weeks gestation or earlier. All 254 households were visited by study enumerators. Households demonstrated a high level of poverty and food insecurity; the mean poverty score was 13 (SD = 8.2), on a scale of 0 to 100, where a lower score is associated with an increased likelihood of living on less than one US dollar per day [20-22]. Over half of households (55.9%) had no access to a latrine; mean food insecurity score was high at 18 (scale 0-27, SD = 5.3).

**Table 1:** Baseline characteristics of mother-infant pairs enrolled in a Zanmi Lasante PMTCT program in rural Haiti*

Infant Characteristic	N = 254
Female gender, N (%)	109 (42.9)
Birth weight < 2.5 kg, N (%)	
Yes	30 (15.3)
No	166 (84.7)
Caesarean delivery, N (%)	
Yes	2 (0.9)
No	228 (99.1)
Birth location, N (%)	
Home	159 (68.8)
Hospital or health facility	71 (30.7)
Other	1 (0.4)
ARV regimen after delivery, N (%)	211 (83.1)
Median birth order, (IQR)	3 (2-5)
Infant nutrition, N (%)	
Exclusive breastfeeding	0 (0)
Mixed feeding	27 (10.6)
Never breastfed	221 (87.0)
Unknown	6 (2.4)
Premature at delivery (< 37 weeks), N (%)	
Yes	13 (5.4)
No or unknown	227 (94.6)
Received food assistance from Zanmi Lasante, N (%)	186 (80.9)
**Mother/Household Characteristic**	**N = 246**
Mean age in years (SD)	33 (7.5)
First received ART, N (%)	
≤ 28 weeks gestation (or ≥ 10 weeks before delivery)	148 (58.3)
28-34 weeks gestation (≥ 4 weeks but < 10 weeks before delivery)	61 (24.0)
< 4 weeks before delivery but before onset labor	40 (15.8)
At delivery	5 (2.0)
Mean Poverty Score (Range 0 to 100^‡^; SD;)	13 (8.2)
Mean Food Insecurity Score (Range 0 to 27, SD)^#^	18 (5.3)
Drinking water source, N (%)	
Improved water source (including filter or purchased water)	129 (56.1)
Poor water source	101 (43.9)
Latrine, N (%)	
Improved latrine	101 (44.1)
No facilities	128 (55.9)
Mean time (hours on foot) to get to ZL clinic (SD)	3.5 (10.91)

* At the time of delivery

‡ A lower poverty score is associated with an increasing likelihood of being a family living on less than 1 US dollar per day

# A higher food insecurity score is associated with worse food insecurity

ART = Antiretroviral therapy

IQR = Interquartile Range

SD = Standard Deviation

ZL = Zanmi Lasante

**Figure 2:**
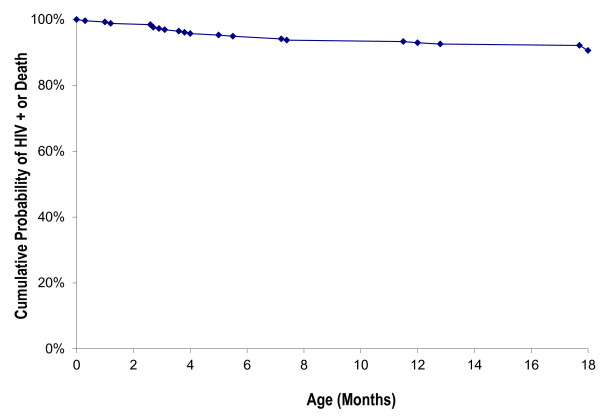
**Cumulative probability of HIV infection or death in a cohort of children in a Zanmi Lasante PMTCT program in rural Haiti**.

At 18 months of age, 9 children (3.5%) were HIV-infected and 17 (6.7%) had died. HIV infection or death occurred in 24 (9.4%). HIV-free survival at 18 months was 90.6% (Figure 2). HIV status and vital status was unknown for 12 infants. When analysis was restricted to infants who were exclusively formula-fed, the overall HIV-free survival rate was 93.7%, and rates of HIV transmission (3.2%) and HIV infection or death (8.6%) were lower. Univariate analysis demonstrated an association at the P < 0.10 level between lower HIV-free survival and low birth weight (P = 0.09), prematurity (P = 0.09) and higher birth order (P = 0.04). On multivariate analysis, however, no factors were found to be significantly associated with survival (data not shown).

For controls, 254 community infants were selected by the method previously described, matched according to gender and age. Table 2 presents the characteristics of each group. Notably, controls differed from PMTCT infants in being more likely to be breastfed (P = 0.003), and mothers were more likely to introduce solid food early (P = 0.003). At baseline, community mothers were more educated (P = 0.04), and more likely to be married or have a civil partnership (P = 0.03), yet were more food insecure (P = 0.02) than their PMTCT counterparts.

**Table 2:** Characteristics of mother-infant pairs in a Zanmi Lasante PMTCT program and matched community controls in rural Haiti**

Infant Characteristic	PMTCT (N = 254)	Community (N = 254)	P-value
Female gender, N (%)	109 (42.9)	123 (51.9)	--^
Mean age (months), (SD)	37 (9.7)	36 (10.7)	--^
Infant nutrition, N (%)			< 0.0001
Exclusive breastfeeding*	0 (0)	75 (31.8)	
Mixed breastfeeding	27 (10.6)	159 (67.4)	
Formula feeding	221(87.0)	2 (0.8)	
Unknown	6 (2.4)	0 (0)	
Mean age (months) other food introduced (SD)	6 (3.0)	5 (4.5)	0.003
**Mother or Guardian Characteristic**	**(N = 246)**	**(N = 250)**	
Mother alive, N (%)	213 (93.8)	209 (92.5)	0.56
Mean age in years (SD)	33 (7.5)	32 (9.1)	0.71
Civil status of mother, N (%)			0.03
Married or civil partnership	166 (71.9)	186 (80.5)	
Divorced, separated, widowed or single	65 (28.1)	45 (19.5)	
Educational Level, N (%)			0.04
None	132 (57.4)	108 (47.0)	
Primary or partial primary	79 (34.4)	88 (38.3)	
Secondary	17 (7.4)	33 (14.4)	
Higher	2 (0.9)	1 (0.4)	
**Household Characteristic**			
Source of drinking water, N (%)			0.99
Poor water source	101 (43.9)	102 (44.2)	
Improved (including ownership of filter)	127 (55.2)	127 (55.0)	
Purchased	2 (0.9)	2 (0.9)	
Mean duration to fetch water (minutes, SD)	28 (35.7)	27 (32.9)	0.7
Latrine			0.89
No facilities	128 (55.9)	130 (56.5)	
Improved latrine	101 (44.1)	100 (43.5)	
Mean time (hours on foot) to ZL clinic (SD)	3.5 (10.91)	2.7 (5.29)	0.31
Mean Poverty Score (SD)	13 (8.2)	13 (9.5)	0.75
Mean Food Insecurity Score (SD)	18 (5.3)	19 (4.7)	0.02

* Exclusive breastfeeding = no formula use, and did not introduce other foods until ≥ 6 months of age

** Unless otherwise indicated, all characteristics correspond to the time of interview

^Matched variables

SD = Standard Deviation

ZL = Zanmi Lasante

In univariate analysis of nutritional status as the dependent variable, there were no significant differences in weight-for-age Z score overall between groups when comparing either median Z score for the groups or analyzing proportions less than 2 or less than 3 standard deviations from the WHO reference score (Table 3). However, in the category of children aged 12-23 months old at the time of survey, median Z score was better in the PMTCT children (-0.08 vs. -0.99, P = 0.03) than in the comparison group. Similarly, the PMTCT cohort in this age group had a trend of fewer children below 2 or 3 standard deviations than the control group, although this was not statistically significant. Community controls demonstrated an almost 2.5-fold increase in risk of diarrheal disease on univariate analysis compared to the PMTCT cohort (30.3% vs. 12.2%; P < 0.0001).

**Table 3:** Univariate analysis of nutrition outcomes comparing infants in a Zanmi Lasante PMTCT program to matched community controls in rural Haiti

Z Score									
	PMTCT Infants			Community Controls			Univariate P value*		
World Health Organization age categories (months)	Median Z Score (SD)	Proportion ≤ 2 SD (95% CI)	Proportion ≤ 3 SD (95% CI)	Median Z Score (SD)	Proportion ≤ 2 SD (95% CI)	Proportion ≤ 3 SD (95% CI)	Median Z Score (SD)	Proportion ≤ 2 SD (95% CI)	Proportion ≤ 3 SD (95% CI)
0-60 (All)	-1.01 (1.32)	25.6 (19.4, 31.8)	7.2 (3.5, 11.0)	-1.10 (1.23)	25.0 (19.1, 30.9)	4.0 (1.2, 6.8)	0.46	0.89	0.14
12-23	-0.08 (1.11)	0.0 (0.0, 4.2)	0.0 (0.0, 4.2)	-0.99 (1.15)	16.0 (0.0, 32.4)	0.0 (0.0, 2.0)	0.03	0.14	n/a
24-35	-1.03 (1.38)	24.2 (14.8, 33.5)	7.7 (1.7, 13.7)	-1.01 (1.33)	23.2 (14.1, 32.2)	5.3 (0.2, 10.3)	0.93	0.87	0.50
36-47	-1.17 (1.33)	33.8 (22.3, 45.2)	8.1 (1.2, 15.0)	-1.34(1.17)	35.2 (23.4, 47.0)	2.8 (0.0, 7.4)	0.41	0.86	0.16
48-60	-0.95 (1.04)	20.0 (4.0, 36.0)	6.7 (0.0, 17.3)	-0.94 (1.12)	15.2 (1.4, 28.9)	6.1 (0.0, 15.7)	0.98	0.61	0.92

* PMTCT compared to community controls

CI = Confidence Interval

SD = Standard Deviation

Table 4 displays multivariate logistic regression analysis for moderate malnutrition (≤ 2SD) and diarrhea outcomes. There was no statistically significant difference in the level of moderate malnutrition (≤ 2SD) between the PMTCT and community group (OR = 1.05; 95% CI: 0.67, 1.64; P = 0.84), after controlling for guardian's education level, marital status, and food insecurity. The PMTCT group fared better with respect to diarrheal disease, with the community group demonstrating a 2.9-fold increased risk of diarrhea compared to the PMTCT group after controlling for confounding factors (95% CI: 1.79, 4.63; P < 0.0001).

**Table 4:** Multivariate logistic regression analysis of malnutrition and diarrhea outcomes in a cohort of infants in a Zanmi Lasante PMTCT program and community controls in rural Haiti

Variable	Outcome			
	Moderate Malnutrition (Z Score ≤ 2 SD)		Diarrhea in the previous 2 weeks	
	Odds Ratio (95% CI)	P-value	Odds Ratio (95% CI)	P-value
Group		0.8447		< 0.0001
Community Control	1.05 (0.67, 1.64)		2.88 (1.79, 4.63)	
PMTCT Infant	1.00		1.00	
Guardian's education level		0.0015		0.8042
None	2.09 (1.33, 3.28)		1.06 (0.67, 1.66)	
Any education	1.00		1.00	
Mother's Civil Status		0.7189		0.6867
Married or civil partnership	1.10 (0.65, 1.88)		0.90 (0.53, 1.53)	
Divorced, separated, widowed or single	1.00		1.00	
Food insecurity score	0.99 (0.95, 1.04)	0.7692	1.07 (1.02, 1.12)	0.0107

CI = Confidence Interval

SD = Standard Deviation

## Discussion

National guidelines for infant feeding in the context of HIV in Haiti are under revision at the time of writing this manuscript, influenced appropriately by the new WHO guidelines [23]. In the context of informing mothers with HIV infection about infant feeding alternatives, we report in this study on the safety of non-breastfeeding for HIV-infected mothers enrolled in a comprehensive PMTCT program in rural Haiti.

### HIV transmission rate and HIV-free survival

Among infants enrolled in Zanmi Lasante PMTCT program, the rate of HIV transmission at 18 months was acceptably low at 3.5%. HIV-free survival in our study was 90.6% at 18 months of age. This rate of HIV transmission is low compared to historical rates of MTCT in Haiti [24]. A non-randomized prospective cohort study in Tanzania using a similar ART regimen from week 34 of pregnancy through 6 months of breastfeeding resulted in an MTCT rate at 18 months of age of 6.0% and HIV-free survival of 86.4% [25]. In Kenya, also using a similar ART regimen in pregnancy through 6 months of breastfeeding, MTCT and HIV-free survival at 18 months were 6.7% and 84.7% respectively. In this and other studies, mother-to-child transmission was lower when maternal viral load was less than 10,000 copies/ml than when baseline viral load was 10,000 or greater [4],. Although HIV viral load testing is not available routinely for mothers in Zanmi Lasante programs in Haiti, treatment is in large part supervised by community health workers called 'accompagnateurs', with high reported rates of ART adherence [26]. This observation of adherence of ART likely contributes to the program's good outcomes. The findings of this current study support those of a Partners In Health/Ministry of Health of Rwanda program study where HIV-free survival was found to be 95% (CI 91-97%) at 12-18 months [27].

We did not find significant predictors of HIV-free survival among the cohort and this is most likely due to the limited size of the cohort and a lack of heterogeneity of key variables among mother-infant pairs. We did however find that among women who never breastfed, HIV-free survival was slightly better than among the cohort overall.

### Risk of non-HIV co-morbidities

Appropriately, one of the key principles in the WHO guidelines in infant feeding in the context of HIV infection is balancing HIV prevention with protection from other causes of child mortality. For HIV-exposed infants in South Africa, Coutsoudis et al. observed a 2-fold increase in risk of morbidity (e.g. diarrhea, lower respiratory tract infection, ear infection) among those who had never breastfed compared those who had reported ever breastfeeding [28]. To assess the impact of the Zanmi Lasante PMTCT program on certain childhood co-morbidities, we measured diarrhea and malnutrition prevalence in the PMTCT cohort and compared those prevalence rates to community controls to establish whether or not the PMTCT program had a negative impact on childhood morbidity in these domains.

In our study, prevalence of diarrhea in the previous two weeks was 30.3% for community controls and 12.2% for PMTCT children. Community controls had a 2.9-fold increased risk of having diarrheal disease in the two weeks prior to the survey compared to the Zanmi Lasante PMTCT group (p < 0.0001). Controls in our study were selected to minimize unmeasured confounding factors related to socio-economic group, sanitation facilities and distance from medical care and we adjusted in multivariate analysis for other factors that were different at baseline. The fact that diarrhea prevalence was significantly lower in the PMTCT group than in the communities where PMTCT mother-infant pairs resided suggests at a minimum that the use of formula feeding by the PMTCT women earlier in the infant's life was not associated with adverse rates of diarrhea in their children by the time of follow up. In the 2005-2006 Demographic and Health Survey, 24% of Haitian children reported diarrhea in the previous two weeks--a statistic that provides further contextual reference for the lower prevalence of diarrhea in the PMTCT cohort in addition to the measured community comparison group prevalence [29]. We propose that education on nutrition and clean water as well as familiarity with health services from early in the infant's life contributed to this lower rate of diarrhea.

We found no statistically significant difference in the PMTCT infants and community controls in terms of moderate or severe malnutrition prevalence. This suggests at a minimum that the PMTCT program did not result in malnutrition. There was a trend amongst children aged 12-23 months to have less moderate or severe malnutrition than community controls. This may be explained by the fact that, recognizing increased vulnerability during this time period, Zanmi Lasante provides a modest form of supplementary food assistance either as a ration or a cash transfer to women in the PMTCT program while their infants are between 9 and 18 months of age.

Zanmi Lasante provides a comprehensive package of child health services in which HIV services are integrated into primary healthcare and women's health programs. This includes access to supplies and training on clean water in addition to training on formula preparation. Other studies support such interventions as resulting in lower diarrheal rates. An observational study of an intervention to promote clean water among HIV-positive women through provision of materials for point-of-use treatment and safe water storage supplies reported a lower risk of diarrheal disease in infants after weaning; however, no reduction in risk was observed during weaning. The authors observed that women demonstrated a high level of adherence to the intervention based on empirical evidence of water testing [30].

These findings on nutrition and diarrhea are supported by other studies on breastfeeding replacement in resource-limited settings. A study in Mozambique, Tanzania, and Malawi compared malnutrition rates among formula-fed and breastfed infants and observed that the prevalence of low weight-for-age Z score estimates was similar in both groups (11.4% and 11.1%, respectively) at six months of age [31]. In Cote d'Ivoire, no differences in risk of validated morbidity occurred among breastfed versus formula-fed infants after two years of follow-up [32]. In that study, although formula-fed infants had slightly higher rates of diarrheal disease and respiratory infection compared with the breastfed group, this did not translate into a higher burden of serious adverse events, such as hospitalization or mortality [32]. These outcomes are likely in good part attributable to the comprehensive services that the study populations received free of charge to support the women's choice of substituting breast milk [6,31].

### Background on Infant Feeding Practice

Avoiding harm to infant feeding practices in the general population is an important component of any PMTCT program. In Haiti, 96% of under-5 year olds are breastfed for an average of 18.8 months; however, background breastfeeding practices are very poor. Infants are exclusively breastfed for a median of 1.5 months and predominantly breastfed for a median of 3.6 months [29]. Breast milk substitution has previously been demonstrated to be acceptable to women with HIV in Haiti [24]. Contrary to concern that recommending breast milk substitution in this targeted population would undermine attempts to reinforce breastfeeding as best practice for the general population ("spillover"), community mothers in our study had higher rates of exclusive breastfeeding than the general population. Among community control mothers, 75% reported exclusively breastfeeding and only 2 women (0.8%) in the community controls reported ever using formula. PMTCT mothers were more likely than their community counterparts to feed exclusively for six months with their method of choice. Although there are indeed improvements to be made in the general population's breastfeeding practices, this shows that there is little to no evidence to suggest that "spillover" is occurring in the Haiti program.

### Limitations

Our study is limited by being an observational study. An effort to minimize confounding was pursued through the selection of comparable children in the same community who were matched by age and gender. In addition, confounding variables were controlled for in the analysis as indicated. Although we were able to study morbidity, given the fact that the study was based on a retrospective analysis we were unable to compare mortality. As surveys are subject to response bias, community participants may have under-reported mixed feeding practices. Because HIV testing is not performed programmatically in the early postnatal period, we were unable to determine the timing of HIV transmission and therefore which component of the PMTCT protocol most contributed to HIV-free survival. The generalizability of the study does not transfer to all medical care in all resource-poor settings as the program specifically attempts to address the social and economic factors associated with ill-health in the patients that are served. However, the generalizability would extend to other resource-poor settings where an effort is made to invest resources in addressing these factors as part of comprehensive healthcare.

### Conclusion

We acknowledge that in many poor settings, resources are not made available to ensure that the safe substitution of breast milk is possible, as we have done in Haiti [33,34]. Research has also now opened the door to other safe alternatives for HIV-infected women who wish to continue to breastfeed their infants--specifically by continuing ART use during breastfeeding. If outcomes were comparable, breastfeeding with ART has a number of benefits as a public health strategy when compared to breast milk substitution. First, it allows for a single message to the population on breastfeeding; second, ART is generally of lower cost than formula and finally, ART plus breastfeeding is easier to implement because it does not require HIV program planners to include water and sanitation improvements in patient care plans and budgets. The latter is a significant challenge for many programs, particularly given the verticality in donor interests and the narrow range of intervention of many 'HIV implementer' organizations.

Despite more than 15 years of evidence of interventions that reduce the transmission of HIV from mother-to-child, 370,000 children were infected with HIV in 2009 [1]. Discussion, debate and research continue on the ideal choice of antiretroviral therapies and the most appropriate public health policies for prevention of transmission from mother-to-child in resource-limited and these are welcomed. Despite this, there remain significant structural, financial and leadership barriers to proper implementation of the tools that already exist and that we already know work [35]. Our data demonstrate that for women who preferred to avoid exposing their infants to breast milk postpartum, providing a safe and feasible environment for breast milk substitution was possible in a PIH-supported program in rural Haiti. This study reflects evidence from a "real-life" program where efforts are made to integrate health services, to provide attention to safe feeding practices for all women and where access to potable water and community education are considered essential components of all public health activities for women, children and families regardless of their HIV-status.

## List of Abbreviations

3TC: Lamivudine; ART: Antiretroviral Therapy; AZT: Azidothymidine; CI: Confidence Interval; HIV: Human Immunodeficiency Virus; NVP: Nevirapine; PMTCT: Prevention of Mother-to-Child Transmission; SD: Standard Deviation; WHO: World Health Organization; ZL: Zanmi Lasante.

## Competing interests

The authors declare that they have no competing interests.

## Authors' contributions

All authors contributed to design of the study. LCI, SCA and JGJ coordinated data collection. BW and MSF performed the statistical analysis. BW, MSF, KAC and LCI helped to draft the initial manuscript. All authors read and approved the final manuscript.
